# The classification of psychiatric disorders according to DSM-5 deserves an internationally standardized psychological test battery on symptom level

**DOI:** 10.3389/fpsyg.2015.01108

**Published:** 2015-08-04

**Authors:** Dalena van Heugten – van der Kloet, Ton van Heugten

**Affiliations:** ^1^Nuffield Department of Clinical Neurosciences, Sleep and Circadian Neuroscience Institute, University of OxfordOxford, UK; ^2^GGZ Oost BrabantBoekel, Netherlands

**Keywords:** psychopathology, DSM-5, classification, categorical vs. dimensional, taxometric method

## Failings of a categorical system

For decades, standardized classification systems have attempted to define psychiatric disorders in our mental health care system, with the *Diagnostic and Statistical Manual of Mental Disorders* (5th ed.; DSM-5; American Psychiatric Association, 2013) and *International Statistical Classification of Diseases and Related Health Problems 10th revision* (ICD-10; World Health Organization, 2010) being internationally best-known.

One of the major advantages of the DSM must be that it has seriously diminished the international linguistic confusion regarding psychiatric disorders. Since its introduction, it contributed extensively toward one common international language for defining and conceptualizing psychiatric disorders. Strikingly, within the field of psychological testing a similar step forward seems to have not yet been taken. At present, there exists no international standard for the use of psychological tests that takes the definition of a specific symptom as listed in DSM-5 as its starting point, and reliably and validly measures this symptom. Rather, the majority of tests are measuring constructs consisting of a multitude of symptoms. For example, the *Beck's Depression Inventory* (Beck et al., 1996) measures core symptoms of depression summing up to a depression score. Accordingly, we believe it is time for a change.

The diagnostics of psychiatric disorders, where disorders are defined as nosological units with a single cause, a single organic substrate, and a single time course, has been problematic for centuries. The field of psychiatry has always been ambivalent about its desire to follow a medical model (Blaney, 2015), but afflicted due to its definitions of pathology. The definition of a psychiatric disorder in DSM-5 offers little room for a clear cut pathogenesis and harsh demarcation of syndromes. This is reflected in the DSM-5, where it states: “A mental disorder is a syndrome characterized by clinically significant disturbance in an individual's cognition, emotion regulation, or behavior that reflects a dysfunction in the psychological, biological, or developmental processes underlying mental functioning” (American Psychiatric Association, 2013, p. 20). An unfortunate and recognized consequence of this definition within the current system is that many symptoms overlap within categories of psychiatric disorders and patients end up diagnosed with many co-morbid disorders.

## Two fundamental problems

Regardless of new attempts to improve the diagnostics of psychiatric disorders, the DSM-5 represents the status quo. Consequently, the distinction between diagnosis and classification remains substantial and more than a discussion on semantics. We identify two fundamental problems within the current framework.

Generally, a psychiatric diagnosis is considered to be descriptive (Hengeveld and Schudel, 2003). The clinician will describe a syndrome (its nature, timespan, and severity) within the framework of a disorder, and often include a differential diagnosis, predisposing protective or vulnerability factors, and provoking or maintaining factors. Within the DSM-5 framework, a similar “approach to clinical case formulation” is taken (American Psychiatric Association, 2013, p. 19). Evidently, a descriptive diagnosis has a hypothetical character. It consists of the clinician's hypothesis and is based on his or her professional considerations according to the abovementioned factors. Once the clinician has formulated a descriptive diagnosis, it is then complementary “translated” into a DSM-5 classification. Herein lies the problem. For the layman, the existence of this distinction between diagnosis and classification is usually unknown. Preceding treatment, the clinician will give the client a classification of the existing psychiatric problems and the client may attribute more meaning to it than appropriate, thereby fostering the risk of reification.

Second, the DSM-5 is a categorical system. Thus, individual disorders are regarded as discrete units—“you either have it, or you don't.” DSM-5 states about this: “(…) scientific evidence places many, if not most, disorders on a spectrum with closely related disorders that have shared symptoms, shared genetic and environmental risk factors (…).” And “(…) we have come to recognize that the boundaries between disorders are more porous than originally perceived” (American Psychiatric Association, 2013, p. 6). This leads to a fundamental problem. Because the overwhelming majority of psychiatric disorders examined thus far using taxometric methods appear to be dimensional in nature (Haslam, 2003; Widiger and Samuel, 2005), consequently all of their categorizations become artificial and debatable. Even though DSM-5 took a modest step toward a more dimensional approach, its core remains categorical.

To illustrate, consider the Borderline Personality Disorder (BPD). This classification consists of nine diagnostic criteria of which a minimum of five need to be present for the diagnosis of BPD. A simple numerical combination algorithm leads to a staggering number of 256 distinct presentations of BPD (Albion et al., 2013). Due to this chameleon-like nature, the disorder's diagnostic validity becomes questionable. Strikingly, this number is relatively small when compared to other conditions, e.g., there are 636,120 ways to have posttraumatic stress disorder (Galatzer-Levy and Bryant, 2013).

DSM-5 does propose an alternative model for personality disorders based on personality functioning and traits (American Psychiatric Association, 2013, p. 761), as a possible answer to the problem that most patients fit with multiple co-morbid personality disorders or to the category of personality disorder not otherwise specified. In November 2012, the chair committee of APA decided to move this alternative model to section III of DSM-5 and to sustain the categorical system in section II.

## A potential solution

DSM-5 and its predecessors have brought about an invaluable improvement regarding the formulation of a common international language for psychiatric disorders. However, the individual disorders fit poorly with its starting point of discrete units and strict boundaries. Van Os (2014) has argued for a better balance between the categorical and personalized aspects of psychiatric disorders. We argue for a two-step approach.

Particularly due to the “weak boundaries” between disorders, it might be beneficial to limit ourselves by merely categorizing clients into the main categories of the DSM-5 according to their most prominent symptoms (Van Os, 2014), i.e., their main complaint. In other words, clinicians could first ask themselves whether the symptomatology is concerning a neurodevelopmental disorder, or a bipolar and related disorder, or a depressive disorder, and so on. It will drastically reduce the over 400 classifications in DSM-5 down to 20 categories. Van Os (2014) even argues to combine some of the main categories, reducing this number even further to 15 categories. The advantages of reducing the over 400 classifications are fivefold: (1) a syndrome, being an aggregation of symptoms, creates a false relation between symptoms that are already heterogeneous themselves; (2) 15 broad categories are functional: it results into a very heterogeneous group of clients within a category and thus prevents stereotypes and invites further personalizing of complaints; and (3) a dimensional measure of a symptom rather than a syndrome will correspond better with the client regardless of it not providing the complete picture. Focusing on the main complaint will indicate where there is an immediate need for care (Van Os, 2014); (4) it will allow clinicians to recognize subthreshold conditions more easily (Magruder and Calderone, 2000); and (5) it might facilitate the development of clear demarcations between normal and abnormal functioning (Kessler, 2002; Widiger and Samuel, 2005). Please, see also Table 1.

**Table 1:** **Comparison of advantages and disadvantages of the dimensional vs. the categorical model**.

**Dimensional**		**Categorical**	
±	More complex, more specific and precise information—i.e., 30 facets by Lynam and Widiger (2001)	+	Presents useful clinical information in succinct manner—one diagnosis (Frances et al., 1995)
+	Decisions for medication or hospitalization often have very different cut-off points. And due to differences in predominant features of disorders for specific patients, these can also vary significantly (Widiger and Samuel, 2005).	+	Decision for medication or hospitalization is categorical—thus specific points of demarcation are needed to guide clinical decisions (Widiger and Samuel, 2005)
+	Internally valid to describe specific patient's psychopathology (Kass et al., 1985)	−	Inaccurate and misleading descriptions (Maser et al., 1991)
+	Less criteria to assess—i.e., smaller set of underlying dimensions of functioning (Haslam, 2002)	−	Thousands of valid categorical distinctions
+	Better able to recognize subthreshold conditions (Magruder and Calderone, 2000)	−	Frequent use of Not Otherwise Specified lacks clinical utility—inadequate diagnostic coverage (Verheul and Widiger, 2004)
+	Avoiding misleading, unstable, illusory effects (Widiger and Samuel, 2005)	−	Confusion—minor changes to a diagnostic criterion often create substantial changes in prevalence rates, further complicating scientific theory and public health policy (Narrow et al., 2002)
+	Potential to facilitate development of clear demarcations between normal and abnormal functioning (Kessler, 2002)		

This also fits well within scientific advances, as thus far strongest evidence has been found for a distinction between internalizing and externalizing disorders, even superseding these 15 main categories (DSM-5; American Psychiatric Association, 2013, p.13, but see Weiss et al., 1998).

The second step would concern the personalized aspect, which we argue to define on a level of core symptoms of the specific main category, to be agreed upon later. To assess these core symptoms, the field of psychological testing could develop an internationally applicable instrument to reliably and validly measure each (core) symptom on a dimensional scale. Many of these instruments are readily available and have proven its psychometric properties within scientific research. Elements from these instruments could be easily transformed to work within the new system. Within the field of psychology, this approach could aid development of a common international language to define symptoms, analogous to the field of psychiatry. Importantly, current psychological testing mainly implies the use of self-report measures. This leads to an abundance of auto-amnestic information. To increase the reliability and validity of psychological tests to assess DSM-5 symptoms, it may be important to add two additional information resources. These would entail psychological tests where a next of kin answers questions about the symptom of the client, and psychological tests that include the clinicians' judgment, such as structured clinician-based interviews. Conversely, we also need to acknowledge the well-known limitations of clinical judgment (e.g., Faust, 1986; Garb, 1998).

## Advantages of a new framework

The changes we suggest have big implications and are not easily implemented in the current framework. Clients may prefer the comfort of a single clear label, similar to what they are used to in other areas of medicine. Health insurance providers and policy makers may argue for single labels as well. Therefore, it would require a lot of explanation from our clinicians to promote a change. Our suggestions for broader categories may not disambiguate the current situation better than the existing approach, but it will promote personalization of health care. Difficulties in diagnostic reliability will remain in disorders that fall on the boundaries of the taxons, e.g., schizoaffective disorder vs. bipolar disorder with psychotic characteristics, which may benefit from further study. There are also some politically tinged questions to keep in mind: How will we finance our health care, and how will we ensure a gradual transition from the current financing system, including clients who already have their labels? What about visitations from insurers and health inspection? How can they control for a good standard of quality in health care when considering the increasing heterogeneity?

Nevertheless, we argue that the benefits outweigh the disadvantages. We will see a shift from a categorical to a personalized approach, which will lead to less (self) stigmatizing, less estrangement, and it will challenge reification thinking. DSM-5 states that “a reformulation of research goals should also keep DSM-5 central to the development of dimensional approaches to diagnosis that will likely supplement or supersede current categorical approaches to diagnosis in coming years” (American Psychiatric Association, 2013, p. 13). Our proposition for psychological testing on symptom level could contribute to this development. Furthermore, this new approach will do more justice to the heterogeneity of symptoms within and outside of classification categories, see also Table 1. Moreover, in this new approach we join forces of expert knowledge from the fields of psychiatry and psychological science. Finally, it offers new and potentially better opportunities to map health care needs. This in turn will lead to a better interface for allocation of appropriate treatment. It will present a clearer picture of when preventative care is preferred over treatment and vice versa. Importantly, health care needs will be more closely attuned to the “own story” of the client.

We argue for the national and international psychological associations in Europe and the United States of America to support the idea of a collective approach to develop an internationally standardized psychological testing battery to reliably assess all the core symptoms of the main categories in DSM-5.

### Conflict of interest statement

The authors declare that the research was conducted in the absence of any commercial or financial relationships that could be construed as a potential conflict of interest.
